# Change of Tricuspid Regurgitation Velocity as a Biomarker for All-Cause Mortality∗

**DOI:** 10.1016/j.jacadv.2023.100576

**Published:** 2023-08-25

**Authors:** Irene M. Lang, Nika Skoro-Sajer

**Affiliations:** Division of Cardiology, Department of Internal Medicine II, Center of Cardiovascular Medicine, Medical University of Vienna, Vienna, Austria

**Keywords:** echocardiography, survival, tricuspid regurgitation

**Figure:**
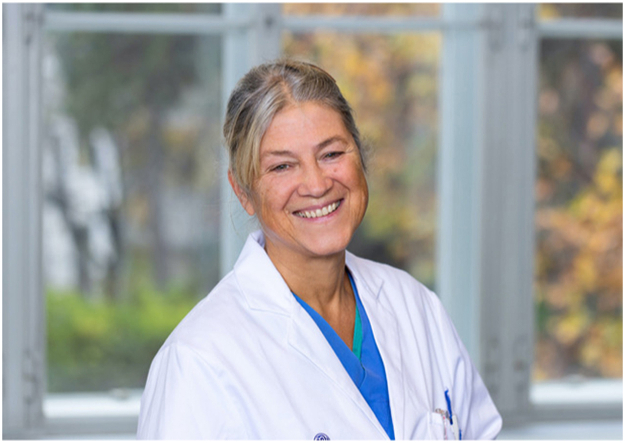

In normal subjects, back flow through the tricuspid valve may occur physiologically.^1^ In a prospectively enrolled cohort of 614 healthy volunteers, mean tricuspid regurgitation velocity (TRV) was 2.01 ± 0.29 m/s (5% to 95% CI: 1.987-2.033 m/s), and significantly but weakly positively correlated with age, body mass index (BMI), systolic blood pressure, diastolic blood pressure, left atrial volume, and negatively with mitral inflow E/A ratio. No differences were found between males and females.^1^ Increasing age is physiologically associated with an increase in TRV, even though regurgitation severity may be trivial to mild.^2^ However, increased vena cava back flow through the tricuspid valve may be a clinical hallmark of dysfunctional right ventricular to pulmonary arterial coupling, or right ventricular to vena cava coupling^3^ and may occur as a consequence of a multitude of conditions. Principally, pulmonary vascular diseases, right ventricular myocardial dysfunction and primary tricuspid valve disease may account for increased TRV. Furthermore, increased TRV may occur whenever right ventricular preload or afterload is increased acutely and transiently, or chronically. Acutely increased TRV may result from intravascular volume loading through fluid administration, due to an increase in osmotic pressure, due to renal dysfunction, or during external chest compression and in systemic disease conditions.

The present study by Kholdani et al^4^ in this issue of *JACC: Advances* aimed to determine the predictors and clinical significance of TRV progression, regardless of the cause. Authors found that older age, depressed left ventricular ejection fraction, diabetes, hypertension, hyperlipidemia, atrial fibrillation, heart failure, and chronic kidney disease were associated with faster progression of TRV, and those with TRV progression of >0.23 m/s/y had an increased risk of all-cause mortality. Although the importance of tricuspid regurgitation (TR)–derived pulmonary artery pressure estimates for survival has long been recognized^5^^,^^6^ and introduced into clinical risk scores,^7^^,^^8^ there are several new messages in this present work.

First, the authors should be commended on taking an effort to look at the tricuspid valve from a global perspective. Elegant modern reviews on the tricuspid valve either take an in-depth and focused look at tricuspid valve anatomy and function, or at modern imaging features,^9^^,^^10^ classifying primary, atrial, ventricular, and device-induced functional TR, putting aside pulmonary vascular disease, systemic disease, and effects of acute loading conditions on the right ventricle.

Second, authors introduce the concept of annual change in velocity as a predictor of outcome, compared with static TR,^6^ adding a new dimension to linear thresholds. Annual progression has been well studied in other valve disease, for example, in degenerative aortic valve stenosis annual progression >3 mm Hg/s/y was associated with worse outcomes.^11^ However, in contrast to aortic valve stenosis, TRV is a less organic but more functional condition that may regress as the underlying cause disappears. Along these lines, in the present study, those who had a decrease in TRV over the study period had a 60% lower risk of all-cause mortality than those with an increase in TRV, which persisted despite identical multivariable adjustment. Study findings applied to patients with low but also with intermediate and high probability of pulmonary hypertension (PH) at baseline. Whether change in TRV will add another layer of precision on risk stratification in the PH risk table,^5^ will have to be analyzed in future studies of patients with invasively confirmed PH.

Third, while the 2022 European Society of Cardiology-European Respiratory Society guidelines for the diagnosis and treatment of PH^5^ formulate echocardiographic probability of PH and recommendations for further assessment based on a TRV of >2.8 m/s, no adjustments were recommended despite a new definition of PH as a mean invasively assessed pulmonary artery pressure of >20 mm Hg, replacing the old mean invasively assessed pulmonary artery pressure threshold of ≥25 mm Hg.^5^ While this appears counterintuitive, the current work demonstrates that absolute TRV >2.8 m/s was associated with worse overall prognosis with mortality rates of 32.6% amongst those with a TRV >2.8 m/s compared with 18.2% amongst those with a TRV ≤2.8 m/s (HR: 2.11; 95% CI: 1.86-2.41; *P* < 0.001). Thus, the uncertainties regarding the echocardiographic threshold may be considered as settled, and the guidelines were correct without any confirmatory data at the time of the publication (guidelines document page 9^5^).

Fourth, the impact of the findings is toward all-cause, and not only cardiovascular mortality. One explanation is that 3,508 individuals of the study (76% of the study population) had a low likelihood of PH at baseline, and therefore presumably a noncardiovascular cause for TR.

There are some limitations to the study that need to be considered. First, more echo studies in sicker patients may have been a bias toward detecting progression. While this bias cannot be entirely avoided, authors noted significant heterogeneity in TRV progression rates from 0.01 m/s/y to 0.8 m/s/y, which speaks to a broad and mixed population sample. In addition, all studies were performed in the ambulatory setting. Second, the data are entirely echo-based and not systematically validated with for example cardiac magnetic resonance studies. Third, an impact of age may not be completely correctable. Fourth, the influence of cardiometabolic disease remains unexplained, but is still of great interest. A higher BMI was associated with a higher baseline TRV, but not with a greater progression rate, for the same baseline BMI. Fifth, we have learned that TRV should not be looked at in isolation, but right atrial and right ventricular size, size of pulmonary artery, and vena cava collapsibility must be taken into account to understand right ventricular performance. Sixth, TR in the context of left heart disease is extremely common today and represents a large burden to patients and healthcare. With the rapid development of transcatheter solutions which have shown safety and efficacy, there is a growing interest in TR. Whether transcatheter intervention will improve mortality will remain to be demonstrated. According to the present work, halting TRV progression may be enough.

## Funding support and author disclosures

Dr Lang has received funding from the Vienna Science and Technology Fund WWTF LS18-090; has relationships with drug companies including AOP-Health, Actelion-Janssen, MSD, United Therapeutics, Medtronic, and Neutrolis; and in addition to being investigator in trials involving these companies, she has relationships that include consultancy service, research grants, and membership of scientific advisory boards. Dr Skoro-Sajer has received compensation for scientific symposia from AOP-Health, Actelion-Janssen, MSD, Cordis, Medtronic, GlaxoSmithKline, and United Therapeutics.
